# Influenza activity in Europe during eight seasons (1999–2007): an evaluation of the indicators used to measure activity and an assessment of the timing, length and course of peak activity (spread) across Europe

**DOI:** 10.1186/1471-2334-7-141

**Published:** 2007-11-30

**Authors:** John Paget, Richard Marquet, Adam Meijer, Koos van der Velden

**Affiliations:** 1 Netherlands Institute for Health Services Research (NIVEL). PO Box 1568, 3500BN, Utrecht, The Netherlands; 2 European Influenza Surveillance Scheme Coordination Centre, PO Box 1568, 3500BN, Utrecht, The Netherlands; 3 Department of Public Health, Radboud University Medical Centre, Geert Grooteplein 10, 6525 GA, Nijmegen, The Netherlands

## Abstract

**Background:**

The European Influenza Surveillance Scheme (EISS) has collected clinical and virological data on influenza since 1996 in an increasing number of countries. The EISS dataset was used to characterise important epidemiological features of influenza activity in Europe during eight winters (1999–2007). The following questions were addressed: 1) are the sentinel clinical reports a good measure of influenza activity? 2) how long is a typical influenza season in Europe? 3) is there a west-east and/or south-north course of peak activity ('spread') of influenza in Europe?

**Methods:**

Influenza activity was measured by collecting data from sentinel general practitioners (GPs) and reports by national reference laboratories. The sentinel reports were first evaluated by comparing them to the laboratory reports and were then used to assess the timing and spread of influenza activity across Europe during eight seasons.

**Results:**

We found a good match between the clinical sentinel data and laboratory reports of influenza collected by sentinel physicians (overall match of 72% for +/- 1 week difference). We also found a moderate to good match between the clinical sentinel data and laboratory reports of influenza from non-sentinel sources (overall match of 60% for +/- 1 week). There were no statistically significant differences between countries using ILI (influenza-like illness) or ARI (acute respiratory disease) as case definition. When looking at the peak-weeks of clinical activity, the average length of an influenza season in Europe was 15.6 weeks (median 15 weeks; range 12–19 weeks). Plotting the peak weeks of clinical influenza activity reported by sentinel GPs against the longitude or latitude of each country indicated that there was a west-east spread of peak activity (spread) of influenza across Europe in four winters (2001–2002, 2002–2003, 2003–2004 and 2004–2005) and a south-north spread in three winters (2001–2002, 2004–2005 and 2006–2007).

**Conclusion:**

We found that: 1) the clinical data reported by sentinel physicians is a valid indicator of influenza activity; 2) the length of influenza activity across the whole of Europe was surprisingly long, ranging from 12–19 weeks; 3) in 4 out of the 8 seasons, there was a west-east spread of influenza, in 3 seasons a south-north spread; not associated with type of dominant virus in those seasons.

## Background

Influenza has an important impact on societies each season. Surveillance data not only provide valuable information on the burden of disease in the population [1,2], but also enables an assessment of whether the vaccine is a good match with the circulating virus [3,4]. Surveillance may help to plan and allocate health care resources and is important for pandemic preparedness [5]. In addition, the surveillance infrastructure can be used to monitor new emerging respiratory diseases, like SARS or avian influenza in humans [6]. Countries in Europe have shared detailed clinical and virological data via the European Influenza Surveillance Scheme (EISS) since 1996 [7]. This collaborative project is partially funded by the European Commission through the European Centre of Disease Prevention and Control (ECDC) and currently includes 30 countries. The scheme covers a total population of about 450 million inhabitants and an area of roughly 12 million square kilometres. EISS collects two types of data on influenza activity each season: 1) clinical and virological data collected by sentinel GPs and 2) virological data from non-sentinel sources. In the present study the EISS dataset was used to characterise important epidemiological features of influenza activity in Europe during eight winters (1999–2007).

In recent seasons there have been indications that influenza activity first appeared in the west of Europe and then moved east across Europe. As an example, during the 2003–2004 season, activity began in Ireland, the United Kingdom, Portugal and Spain in early August and reached Poland in February 2004 [8]. These observations led us to assess three related questions:

1. Are the sentinel clinical reports a good measure of influenza activity?

2. How long is a typical influenza season in Europe?

3. Is there a west-east spread and/or south-north spread of influenza in Europe?

## Methods

### General

Data from a median of 17 (14–28) countries from the EISS database were analysed for eight influenza seasons: 1999–2000 (14 countries); 2000–2001 (15 countries); 2001–2002 (16 countries); 2002–2003 (17 countries); 2003–2004 (22 countries); 2004–2005 (22 countries); 2005–2006 (22 countries) and 2006–2007 (28 countries). Countries were included in the analysis if they were at least 5 years active member of EISS and if weekly data were available for the full season. The assessment of influenza activity presented in this paper is largely based on data reported by sentinel GPs. The GPs report clinical cases of influenza-like illness (ILI) and/or acute respiratory infection (ARI) to a central registry and take respiratory specimens that are sent to a national reference laboratory for testing. This ensures that the clinical data reported by the sentinel physicians are validated by virological data on influenza. The national reference laboratories also report laboratory test results on non-sentinel respiratory specimens e.g. specimens from hospitals or non-sentinel physicians. These data were collected to have an additional indicator of influenza activity and to validate the sentinel virological data. The national reference laboratories participate in the 'Community Network of Reference Laboratories for Human Influenza in Europe' (CNRL), which is coordinated by EISS [9]. CNRL works closely with the WHO through its network of National Influenza Centres and collaborates with the Centre for Reference and Research on Influenza at Mill Hill, London, UK.

In the current study only the time points of highest clinical and virological activity were used, because only peak levels represent undisputable markers of activity in a given country. The reason not to take whole incidence curves into account is that incidence rates in Europe vary considerably, because: 1) case definitions are not yet harmonised across Europe; most countries report cases of ILI, but some report cases of ARI, 2) the denominator calculations vary by country and, 3) consultation rates for ILI and ARI vary among countries. They not only depend on cultural factors, but also on the delivery of health care. For example, in some European countries a doctor's certificate is required for a single day of absence from work (leading to a higher consultation rate), whilst in others a certificate is only required after absence of 5 days or more, leading to a lower consultation rate.

The weeks of peak activity were selected by plotting the clinical and virological data available for each country. If the clinical and virological activity was very low during a season (e.g. below or around the baseline level; defined as level of influenza activity in the period when no influenza virus was detected), it was difficult to identify the peak week and no peak was selected. Most countries reported cases of ILI to EISS (13 out of the 17 countries); four countries used the less restrictive case definition of ARI (Czech Republic, France, Germany and Romania). Since 2004 the Czech Republic and Romania also report cases of ILI [10]. The case definitions of ILI and ARI have been described by EISS and discussed by Aguilera et al. [11,12]. Briefly; the general criteria for ILI are: sudden onset of fever > 38°C, with respiratory (i.e. cough, sore throat) and systemic symptoms (headache, muscular pain); the criteria for ARI are: sudden onset of respiratory symptoms, accompanied by fever and headache in the absence of other diagnosis.

### Validity analysis

For the validity analysis of sentinel reports, we defined a good match as a situation where the sentinel and virological peaks occurred in the same week, or when there was a difference of only one week. For example the peak of the incidence of ILI consultations in the Netherlands during the 2002–2003 season was week 10 and the peak of positive laboratory reports of the dominant influenza virus was week 9. This difference of 1 week was considered to be a good match. Therefore, a time difference between peaks of 1 week or less was taken as a measure for a good match (irrespective of which peak presented first); a difference of 2 weeks was taken as a reasonable match and a longer period as a poor match. The analysis was based on the percentage of countries fulfilling the criteria of a good or moderate match during 8 influenza seasons. In a second validity analysis, using similar criteria, the sentinel clinical incidences were compared with non-sentinel laboratory reports. Because the case definitions of ILI and ARI differ considerably, the validity analyses were performed separately for countries using ILI (n = 13) and countries using ARI criteria (n = 4). Differences in matching percentages between ILI and ARI were statistically evaluated using the Chi-square test or Fisher exact test if the expected value in one of the cells was less than five.

### Length of influenza season

The length of an influenza season was roughly calculated by subtracting the earliest and latest week of peak clinical activity across Europe for each season. Per season the aggregated data of participating countries were used. Knowing that using peak weeks as indicator for activity would lead to underestimation of the length of the epidemic, because periods of high activity at the beginning and the end were not taken into account, 4 weeks were added: 2 weeks before the earliest peak and 2 weeks after the last peak. These periods still represent a rather conservative estimate of the slopes of increased activity around the incidence peaks. Eight countries participated throughout the 8 seasons (their longitudes ranging from -4 to 15.3); 6 countries during 7 seasons (longitudes -8 to 19.3) and 14 countries were included during less than 7 seasons (longitudes -3 to 25).

### Spread of influenza (course of peak activity across Europe)

The sequence of peak activity of influenza in the various European countries was taken as a measure for the spread of influenza across Europe. We are well aware that the use of the word 'spread' is based on the liberal assumption that the sequence of peak activity across Europe parallels the actual spread of influenza. Therefore, as we have no clear insight into the dynamics of influenza between countries, in the present study 'spread' should be appreciated with some caution and in a very general context. In order to assess a possible west-east spread or a south-north spread of influenza activity in Europe, the peak week data of influenza activity in EISS countries were plotted against the longitude and latitude of the central point in each country. For the purpose of finding the appropriate geographic centre of a country, rounded longitude and latitude figures were used, based on The Gazetteer of Conventional Names, third Edition, August 1988 [13]. For Northern Ireland, Scotland, Wales and England such central points were not available. Therefore we took the capital cities of these regions: Belfast, Edinburgh, Cardiff and London as best substitutes. Considering it was difficult to identify a peak during seasons of low influenza activity, the sentinel virological data, if available, were used to select the peak. If no sentinel virological data were available, no peak was selected.

Regression analysis and analysis of significance was performed using SPSS 11.5. The variance was expressed as squared correlation coefficients (R^2^), interpreted as follows: < 0.1 very weak correlation; 0.1–0.25 weak; 0.25–0.50 moderate; 0.5–0.75 strong; 0.75–0.9 very strong; > 0.9 exceptionally strong correlation.

## Results

### Validity analysis

Table 1 presents the clinical data in 17 countries during eight seasons. When using the norm of +/- 1 week, the mean total overlap of sentinel clinical and virological data was 72% (median 71%; range 25%–100%) for countries using ILI as case definition, and 71% (median 73%; range 57%–83%) for countries using ARI as case definition. When using the norm of +/- 2 weeks, the mean overlap for ILI-countries rose to 84% (median 86%; range 50%–100%) and rose to 85% (median 86%; range 71%–100%) for countries using ARI as case definition.

**Table 1:** Comparison between clinical and virological peak incidences of ILI and ARI during eight influenza seasons in Europe (1999–2007)

	**S. clinical vs. S. virological**	**S. clinical vs. S. virological**	**S. clinical vs. NS. virological**	**S. clinical vs. NS. virological**
	**ILI**	**ARI**	**ILI**	**ARI**

1 week overlap	72%	71%	63%	46%
2 weeks overlap	84%	85%	84%	68%

S: Sentinel, information provided by sentinel general practitioners

NS: Non-sentinel, data obtained from non-sentinel physicians, hospitals and institutions

ILI: influenza-like illness used as case definition by 13 countries

ARI: acute respiratory disease used as case definition by 4 countries

1 week overlap: occurrence of peak incidences differ 1 week or less (good match)

2 week overlap: occurrence of peak incidences differ 2 weeks or less (moderate match)

Percentages represent the mean overall match aggregated from data of most participating countries obtained during 8 influenza seasons

Table 1 also compares the sentinel clinical data with the non-sentinel virological data. When using a match of +/- 1 week, the mean overlap of sentinel clinical and non-sentinel virological data for countries using ILI was 63% (median 67%; range 0%–100%), and 46% (median 50%; range 43%–50%) for countries using ARI. When accepting a match of +/- 2 weeks, the overlap rose to 84% for ILI (median 86%; range 50%–100%), and to 68% for ARI (median 60%; range 50%–100%). The differences between ILI and ARI were statistically not significant (1-week match, p = 0.26; 2-week match, p = 0.19).

### Length of epidemic and dominant virus

Table 2 provides an indication of the length of each influenza season in Europe. They ranged from 12 weeks in the 1999–2000 season to 19 weeks in the 2003–2004 season (mean 15.6 weeks; median 15 weeks).

**Table 2:** Duration, spread and dominant viruses in Europe during eight influenza seasons (1999–2007)

**Season**	**Duration**	**W-E (R^2^)**	**S-N (R^2^)**	Dominant virus(es)**
1999–2000	12 weeks	0.073	0.058	A(H3N2)
2000–2001	18 weeks	0.016	0.013	A(H1N1)/B
2001–2002	15 weeks	0.331*	0.481*	A(H3N2)/B
2002–2003	15 weeks	0.428*	0.005	A(H3N2)/B
2003–2004	19 weeks	0.598*	0.001	A(H3N2)
2004–2005	18 weeks	0.680*	0.250*	A(H3N2)
2005–2006	14 weeks	0.032	0.002	A(H3N2) A(H1N1) B
2006–2007	14 weeks	0.060	**0.287**	A(H3N2)

Spread of influenza based on analysis of peak levels of clinical activity

W-E: West-East spread; S-N: South-North spread

R^2^: squared correlation coefficient (0.250; 0.331; 0.428; 0.481: moderate correlation; 0.598 and 0.680: strong correlation)

*P < 0.05

**: virus (sub)types are only listed if they represented > 20% of total detections

The dominant viruses (viruses most frequently identified in specimens) in Europe during the eight seasons were: influenza A(H3N2) in 1999–2000; influenza A(H1N1) and B in 2000–2001; A(H3N2) and B in 2001–2002; A(H3N2) and B in 2002–2003; A(H3N2) in 2003–2004; A(H3N2) in 2004–2005; influenza B, A(H3N2) and A(H1N1) in 2005–2006 and A(H3N2) in 2006–2007.

No correlation was found between length of the epidemic and type of virus or virus combinations.

### Course of peak activity across Europe

**Figure 1:**
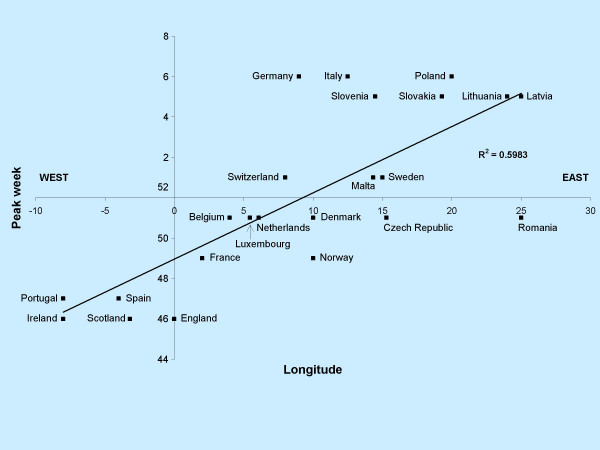
Evidence of West-East spread of influenza in Europe during the 2003–2004 season: the longitude of 23 countries correlated against the peak week of influenza activity per country.

Table 2 assesses the west-east and south-north spread of influenza activity in Europe after respectively plotting the longitude and latitude of each country to the peak clinical level of activity for each season. As an example the plot of the 2003–2004 season is shown in Figure 1. The assessment was based on calculation of the squared correlation coefficient. In four consecutive seasons (2001–2005), there was a moderate to strong correlation between longitude and peak influenza activity, indicating a west-east spread of influenza. It is noteworthy that during these four seasons A(H3) was the dominant virus. However, this was also the case during the 1999–2000 and 2006–2007 seasons, in which there was no indication for a west-east spread. We repeated the analysis for the latitudes of each country and found a moderate correlation in three season, favouring a south-north spread. During two of these seasons (2001–2002 and 2004–2005) there was also a clear west-east spread of influenza. Taking all eight seasons into account the overall indication for a west-east spread was twice as strong than for a south-north spread (mean R^2 ^west-east: 0.277; mean R^2 ^south-north: 0.137).

## Discussion

In the present study routinely collected surveillance data were used to assess the timing, length and spread of influenza activity in Europe during eight winter seasons. It first tried to establish the validity of the sentinel reports and then used these data to assess the length of an average season in Europe and whether or not there is a general west-east spread of influenza activity.

### Validity of sentinel reports

The analysis in this paper is largely based on clinical sentinel reports and it was therefore important to validate this data source, first with the virological sentinel data and then with non-sentinel laboratory data. We found that the data were valid, irrespective of whether ILI or ARI was used as case definition. There was an overall match of 72% (+/- 1 week) between the clinical and virological sentinel data and a 60% match (+/- 1 week) between sentinel clinical data and the non-sentinel virological data. Allowing a larger overlap (+/- 2 weeks) provided a match of 84% and 80%, respectively. The results indicate that the sentinel system is very adequate in estimating influenza activity in a continent, irrespective of case definition. The strength of the system is that it combines community-based clinical and virological data and also can provide age-specific data. The sentinel approach has already been implemented in other continents, our results imply that it should be considered in other parts of the world [14].

It is not surprising that there was a close relationship between the clinical and virological sentinel data. This relationship should be close, because the diagnosis of ILI or ARI and the subsequent collection of respiratory specimens is done by the same person (sentinel GP). Sampling of specimens is also usually at its highest during the period of increased influenza activity, which will lead to increased numbers of positive specimens. Some of the non-matches of sentinel data occurred during the Christmas/New Year period when the clinical and virological surveillance systems are affected by holidays.

The second comparison we performed was between the sentinel clinical and the non-sentinel virological data. This was an important validity check because two independent surveillance systems were compared. Many countries (e.g. the U.S.) do not collect sentinel virological data, but base the virological assessment of influenza activity solely on data from non-sentinel sources [15]. Our study found a good match between sentinel clinical and non-sentinel virological sources. Again, the clinical rates can sometimes be unreliable during seasons when activity is very low, which may lead to mismatches. On the other hand, a large outbreak in a major hospital or region may be another important factor that can affect the non-sentinel data. Such localized outbreaks may lead to increased non-sentinel reports that are not picked up by the sentinel clinical reports, resulting in a mismatch. Taken together, we do favour the sentinel collection of virological data because the approach is more systematic, less prone to pre-selection and differs less among countries than clinical sampling.

### Length of an average season

The mean length of a typical influenza season in Europe based on the peak activity levels of ILI/ARI was 15.6 weeks. It is a conservative estimate, because it does not include the period of increased influenza activity. If this period is also taken into account the average European influenza season lasts about 4.5 months. This is a important finding as it highlights the fact that influenza activity occurs for a long period of time in Europe each season. It also highlights the need to present country-specific data, in order to get insight into the diversity of activity [16].

### Course of peak activity (spread) across Europe

Because the spread of influenza depends of many factors one might not expect a particular pattern. Still, four out of eight seasons showed a clear west-east spread of influenza. In three seasons there was a south-north spread. The overall indication for a west-east spread was stronger than for a south-north spread, which means that a west-east spread of influenza is a more common, but far from consistent phenomenon in Europe. It is noteworthy that the west-east spread occurred in 4 consecutive seasons during which the more virulent A(H3) was the dominant virus. It should be noted that the data for 1999–2000 and 2000–2001 were not complete: a number of important countries to the east of Europe (Latvia, Lithuania, the Slovak Republic and Poland) were not included in the analysis because data were not available. This might have affected our west-east analysis.

In an analysis of the spread of influenza in the US over 30 years, Viboud et al. observed a consistent early onset of the epidemic in California, which is the most populous state in the U.S. [17]. Interestingly, the onset in California was earlier than in 3 populous Eastern states, suggesting that in addition to population factors also geographical and climate factors, such as:, mountain ranges, plains, lakes, and predominant wind direction may drive early epidemic activity. Some of these factors may be involved in a west-east spread in Europe: the western of Europe is the most populated part and factors that contribute to spread, such as commuting and airline travel consequently are more intense in the western than in the eastern part of Europe [18].

We have used a rather crude method to assess the geographic spread of influenza activity in Europe. A more refined method would be to collect doctor specific data as routinely is done in France [19]. As these data are not available yet within EISS we had to use the methodology of analysing peak levels of activity in each country. In 2005 EISS initiated a European Mapping Project, in which data from Germany and the Netherlands were brought together to map the spread of influenza on a weekly basis, using data from about 500 physicians in Germany and 84 in the Netherlands [20]. This project has now been extended to 7 countries and hopefully the upcoming data will allow us to better assess the spread of influenza [21]. These results have important consequences for public health: it allows better planning of health care resources at a local level, and at a European level better recommendations can be made about the timing of vaccination.

## Conclusion

Our analysis has demonstrated that the sentinel clinical data, the main indicator used to measure influenza activity in Europe, is a valid indicator for influenza activity. We also found that the length of influenza activity in Europe was surprisingly long. Finally, during 4 out of 8 seasons there was a clear indication of a west-east spread and in 3 seasons a moderate indication of a south-north spread of influenza activity across Europe.

## Competing interests

The author(s) declare that they have no competing interests.

## Authors' contributions

JP performed the analysis of the data and drafted the manuscript.

RM revised the manuscript and did supplementary analysis.

AM participated in the design of the study.

KV participated in the design and coordination of the study.

They all approved the final version of the manuscript.

## Pre-publication history

The pre-publication history for this paper can be accessed here:


